# RMD-3 and RMD-6 identified as new components of the MSP fibrous body complex in *C. elegans* spermatocytes

**DOI:** 10.17912/micropub.biology.001687

**Published:** 2025-07-28

**Authors:** Nirvanjyoti Sharma Shimul, Elena B. Benson, Jake P. Diaz, Lynn Zavada, Diane C. Shakes

**Affiliations:** 1 Biology, William & Mary, Williamsburg, Virginia, United States

## Abstract

The RMD (regulator of microtubule dynamics) protein family is conserved across various species but their activity and role in spermatogenesis is largely unexplored. Here we report developing an antibody against the two nearly identical proteins *C. elegans* RMD-3 and RMD-6 . This antibody detects RMD-3 /6 in both immunocytology and western blots. RMD-3 /6 colocalizes with the major sperm protein MSP to the fibrous bodies of spermatocytes, cytoplasm of spermatids, and pseudopods of spermatozoa. RMD-3 /6 are sperm-specific, yet male fertility in triple *rmd-2 / rmd-3 / rmd-6* knockout mutants is unaffected under conditions tested.

**Figure 1. RMD-3/6 antibody validation and stage-specific labeling patterns:**
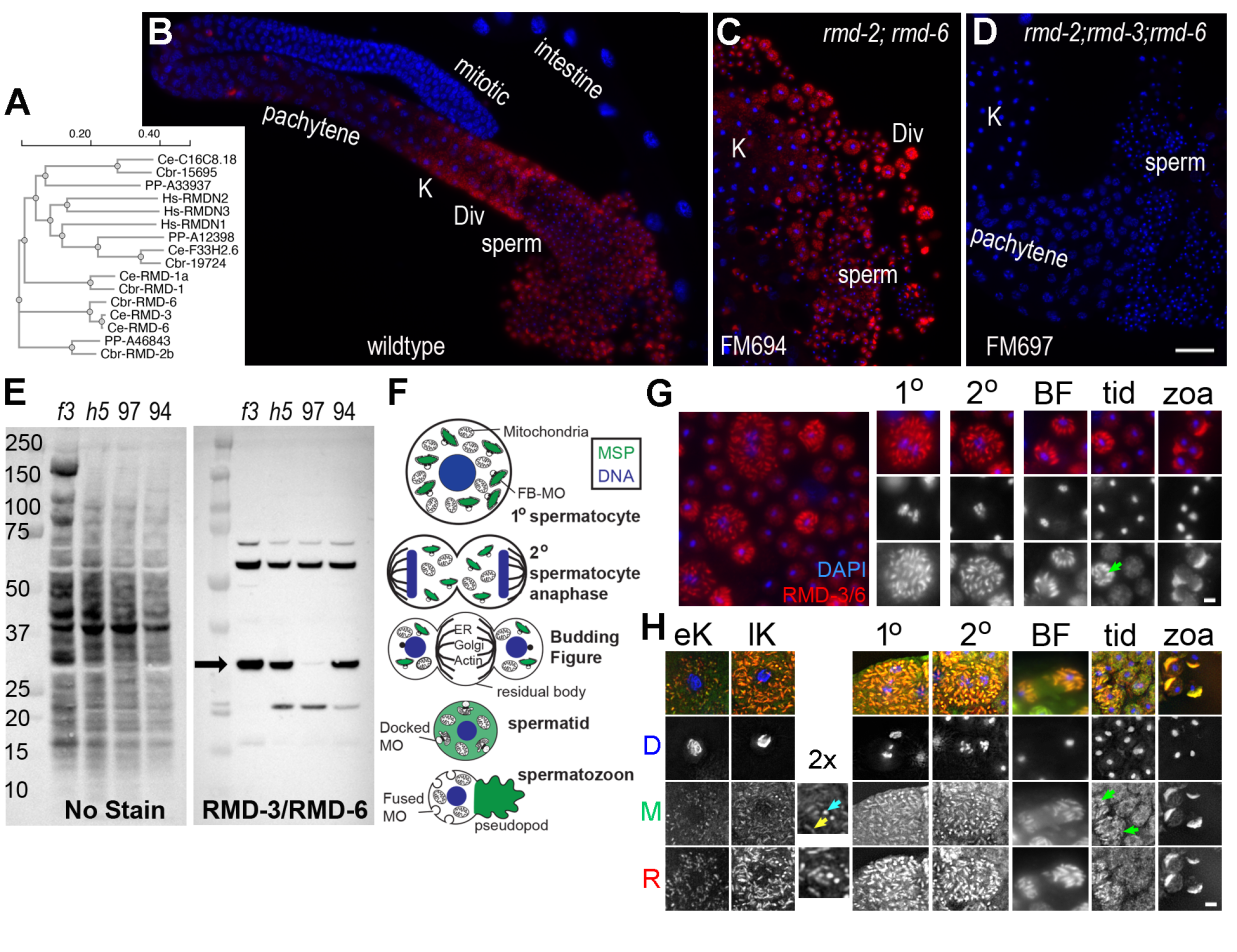
(A) Molecular phylogeny of RMD and RMDN related sequences from humans (Hs), *C. elegans* (Ce), *C. briggsae* (Cbr) and a non- *Caenorhabditis* nematode *Pristionchus pacificus* (PP). The sequences were downloaded from Wormbase WS297, and the phylogenetic analysis was done using Clustal Omega (B-D) Isolated male gonads from wildtype and knock-out mutants co-labelled with DAPI (blue) and anti- RMD-3 /6 antibody (red). (B-D) Scale bar = 10 microns. (E) Western blot of anti- RMD-3 /6 antibody in sperm-only *fem-3* ( *q20* ) hermaphrodites and “wildtype” *him-5* and *rmd-2* ; *rmd-3* ; *rmd-6* (FM697), and *rmd-2* ; *rmd-6* (FM694) males. Arrow indicates RMD-3 /6 band. (F) Cartoon of the meiotic and post-meiotic stages of *C. elegans* spermatogenesis with MSP patterns in green (G) RMD-3 /6 (red) labelling patterns in staged spermatocytes and sperm co-labelled with DAPI (blue). (H) Co-localization of RMD-3 /6 and MSP of staged spermatocytes and sperm. All images except the budding figure (BF) are deconvoluted max Z images. In 2X magnified image, arrows mark the FBs in longitudinal (yellow) and cross-sectional (cyan) orientations. (G-H) Abbreviations: early and late karyosomes (eK, lK), primary and secondary spermatocytes, budding figures (BF), spermatids (tid), and spermatozoa (zoa). Green arrow indicates newly budded immature spermatid. (G-H) Scale bar = 2 microns.

## Description

Conserved between *C. elegans* and humans, members of the RMD protein family are named for their role of both *C. elegans* and human RMD-1 in regulating microtubule dynamics (Oishi et al., 2007; Fig. 1A ). However, RMD proteins also function in non-microtubule roles. For example, human RMD-3 /PTPIP51, localizes to the mitochondrial outer membrane where it facilitates inter-organelle binding interactions with the ER-membrane protein VAPB (De Vos et al., 2012; Mórotz et al., 2022). FFAT motifs and a coiled coil domain within the extended N-terminal domain of RMD-3 mediate binding to the MSP-domain of VAPB, named for its homology to the nematode major sperm protein. Although human RMD-3 /PTPIP51 (Stenzinger et al., 2005) and *C. elegans* RMD-3 /6 proteins (Ma et al., 2014) are both highly expressed in sperm, their sperm functions have yet to be described.

The functions of *C. elegans* proteins RMD-2 /3/6 have not been well studied, but triple knockout (KO) males produce fertilization-competent sperm and hermaphrodites have similar fertility rates to control worms (Juanico et al., 2021). To directly test the fertility of *rmd-2 ; rmd-3 ; rmd-6* triple KO males, we crossed single L4-stage males with three L4-stage *fog-2* “feminized” hermaphrodites which lack self-sperm. To assess full broods, adults were transferred to fresh plates daily. Control males sired an average of 647 +/- 21.8 (S.E.) viable progeny (n=5), and triple deletion males sired an average of 612 +/- 21.8 (S.E.) progeny (n=5), indicating that RMD-3 /6 are not required for sperm function under the conditions tested.

Despite the lack of a knockout phenotype, both the abundance of RMD-3 /6 within sperm and a potential connection to MSP remained intriguing. In sequence comparison studies, we found that RMD-3 and RMD-6 are 98% identical (221/225) and 99% conserved. The molecular phylogenetic tree generated by Clustal Omega (Madeira et al., 2024) suggests that RMD-3 and RMD-6 arose from a recent gene duplication. Named *C. elegans* RMD proteins ( RMD-1 , 2, 3, 6) proved to be more similar to each other than to their human RMDN counterparts. Other *C. elegans* proteins ( C16C8.18 and F33H2.6 ) may be closer homologs of the human proteins ( Fig. 1A ). The smaller *C. elegans* RMD-3 /6 proteins lack the experimentally verified MSP binding domains within the N-terminal domain of human RMD-3 /PTPIP51, yet it remains possible that the *C. elegans* proteins have non-canonical MSP binding sequence motifs that have yet to be identified.

To better understand potential RMD-3 /6 functions, we generated an affinity purified, rabbit polyclonal antibody against a shared peptide sequence. In wildtype male germlines, RMD-3 /6 was first detected in mid-pachytene spermatocytes ( Fig. 1B ). Similar patterns were observed in the germlines of *rmd-2 ; rmd-6* double KO males ( Fig. 1C ) whereas no labelling was observed in germlines of triple KO males ( Fig. 1D ). In western blots, the antibody bound robustly to a protein that migrated slightly slower than the predicted 25.9 kD molecular weight band that was present in sperm-producing *fem-3* (gf) hermaphrodites, *him-5* males, and *rmd-2 ; rmd-6* males, but not in triple KO males ( Fig. 1E ). Although these experiments only directly confirm that our antibody recognizes RMD-3 , it is likely that the antibody recognizes both RMD-3 and RMD-6 as the identical peptide antigen is present in both proteins, and single-cell RNA seq data indicate that both proteins are expressed at high levels during spermatogenesis (Ghadder et al., 2023). Our finding, that both the immunofluorescence and western blot signals remain elevated in *rmd-2* ; *rmd-6* males, suggests either that RMD-6 is expressed at much lower levels than RMD-3 or that these males compensate by upregulating the expression of RMD-3 . Within primary and secondary spermatocytes, RMD-3 /6 antibodies labelled discrete structures throughout the cytoplasm that looked similar to that of MSP-containing fibrous bodies (FB) ( Fig. 1F, G). RMD-3 /6 subsequently segregated to spermatids in post-anaphase II budding figures (BF), dispersed through the cytosol in spermatids, and localized to the pseudopods of spermatozoa. Because the observed localization patterns suggested that RMD-3 /6 was co-localizing with MSP throughout spermatogenesis, we co-labelled male gonads with anti-MSP and anti- RMD-3 /6 antibodies ( Fig. 1H ). Tight colocalization of RMD-3 /6 and MSP was observed at all stages of spermatogenesis. Notably, colocalization was observed in both longitudinal and cross-sectional views of FBs. As previously observed for other sperm proteins, the antibody labeling was brighter in newly budded spermatids than mature spermatids. In newly budded spermatids, MSP and RMD-3 /6 showed tight co-localization to the still intact FBs. In mature spermatids, MSP and RMD-3 /6 dispersed through the cytoplasm but their patterns showed only partial co-localization. In spermatozoa, RMD-3 /6 co-localized with MSP in the pseudopods.

This study adds RMD-3 /6 to the growing list of proteins that are packaged together with MSP in the FBs of developing spermatocytes and ultimately segregate to the pseudopods of motile spermatozoa (Morrison et al., 2021). Although triple KO males were fully fertile under conditions tested, future studies of RMD-3 /6 binding partners promise to reveal new insights regarding the role of RMD proteins in both *C. elegans* and mammalian sperm.

## Methods

*Worm Culture* Worms were cultured on MYOB plates (Church et al., 1995) and inoculated with the *E. coli* strain OP50 , using methods similar to those described by Brenner (1974).

*Fertility Assay* For the fertility analysis, three L4-stage *fog-2* “feminized” hermaphrodites were crossed to single L4-stage control or FM697 triple deletion males on single-spot 35 mm plates. All four worms were transferred to fresh plates daily until there were no more embryos produced. The total progeny sired by individual males was calculated from the sum of the progeny it sired over multiple days.

*Antibodies and Immunochemistry* Sperm spreads were obtained by dissecting 8-10 males per slide in 8 microliters of sperm media on ColorFrost Plus slides (Fisher Scientific, 12-550) coated with poly-L-lysine (Sigma Aldrich, P8290). Slight pressure was applied to coverslips to flatten out the samples. The samples were freeze-cracked in liquid nitrogen and fixed in −20°C methanol overnight. Specimen preparation and antibody labeling followed established protocols (Shakes et al., 2009). Antibodies used included 1:1500 4D5 mouse anti-MSP monoclonal, 1:800 rabbit anti- RMD-3 /6 polyclonal, 1:300 Alexa Fluor Plus 555 goat anti-rabbit IgG, and 1:150 Alexa Fluor 488 goat anti- mouse IgG (H+L). Affinity purified rabbit antiserum against a shared RMD-3 and RMD-6 epitope was generated by YenZym. Slides were mounted with Fluoro Gel with DABCO (Electron Microscopy Sciences #17985-02) containing DAPI.

*Imaging and Analysis* Images were acquired with epifluorescence using an Olympus BX60 microscope equipped with an Orca Flash 4.0LT3 camera. The imaging software cellSens was used to capture, merge, and in some cases capture Z-stacks and deconvolute the images. For control studies ( Fig. 1B- D), image exposures were kept constant with no further imaging processing. For Fig. 1G, image exposures were optimized for individual gonads, and the levels adjust function in Adobe Photoshop was used to spread the data containing regions of the image across the full range of tonalities.

*Western Blot* For western blot analysis, 100 young adult male worms ( *him-5* , FM697, and FM694) were collected in 10 μL of M9 buffer, flash-frozen in liquid nitrogen, and stored at -80°C. Samples were run on an SDS-PAGE gel and transferred to a PVDF membrane (Transblot Turbo, BioRED). Anti- RMD-3 /6 antibody was used at a 1:1,000 dilution. HRP-conjugated goat-anti-rabbit secondary antibody was used at a 1:2,000 dilution, and the blot was visualized using an iBright chemiluminescent imaging system.

## Reagents

**Table:** 

**Strain**	**Genotype**	**Available From**
CB4088	*him-5* ( *e1490* ) V	CGC
JK816	*fem-3* ( *q20* ts)	CGC
JK574	*fog-2* ( *q71* )	CGC
FM697	*rmd-2* ( *du8* ); *rmd-3* ( *tm2635* ); *rmd-6* ( *du9* ); *him-5* ; sdhc-1::mCherry	F. McNally lab
FM694	*rmd-2* ( *du8* ); *rmd-6* ( *du9* ); *him-* 5; sdhc-1::mCherry	F. McNally lab
**Antibody**	**Animal and clonality**	**Description**
Anit-MSP	Mouse monoclonal, 4D5	Kosinski et al., 2005
Anti- RMD-3 /6	Rabbit polyclonal	Rabbit antisera was generated by immunizing a rabbit against the aa 7-20 (DKTFGTKNRDQGYD with an added amide-C) which is common to *C. elegans* B0491.3 RMD-3 and R13H9.1 RMD-6 .
HRP, secondary	anti-rabbit IgG	Abcam
Alexa Fluor Plus 555, secondary	Goat anti-rabbit IgG	Invitrogen, A32732
Alexa Fluor 488, secondary	Goat anti- mouse IgG	Jackson Immunoresearch, 115-545-146
